# Establishment and validation of a prognostic signature for lung adenocarcinoma based on metabolism‐related genes

**DOI:** 10.1186/s12935-021-01915-x

**Published:** 2021-04-15

**Authors:** Zhihao Wang, Kidane Siele Embaye, Qing Yang, Lingzhi Qin, Chao Zhang, Liwei Liu, Xiaoqian Zhan, Fengdi Zhang, Xi Wang, Shenghui Qin

**Affiliations:** 1 Institute of Pathology, Tongji Hospital, Tongji Medical College, Huazhong University of Science and Technology, Wuhan, 430030 China; 2 Department of Pharmacy, Hiser Medical Center of Qingdao, Qingdao, 266033 China; 3 Department of Pathology, Wuhan Third Hospital (Tongren Hospital of Wuhan University), Wuhan, 430030 China

**Keywords:** Lung adenocarcinoma, Metabolism‐related genes, Prognostic, The Cancer Genome Atlas

## Abstract

**Background:**

Given that dysregulated metabolism has been recently identified as a hallmark of cancer biology, this study aims to establish and validate a prognostic signature of lung adenocarcinoma (LUAD) based on metabolism-related genes (MRGs).

**Methods:**

The gene sequencing data of LUAD samples with clinical information and the metabolism-related gene set were obtained from The Cancer Genome Atlas (TCGA) and Molecular Signatures Database (MSigDB), respectively. The differentially expressed MRGs were identified by Wilcoxon rank sum test. Then, univariate cox regression analysis was performed to identify MRGs that related to overall survival (OS). A prognostic signature was developed by multivariate Cox regression analysis. Furthermore, the signature was validated in the GSE31210 dataset. In addition, a nomogram that combined the prognostic signature was created for predicting the 1-, 3- and 5-year OS of LUAD. The accuracy of the nomogram prediction was evaluated using a calibration plot. Finally, cox regression analysis was applied to identify the prognostic value and clinical relationship of the signature in LUAD.

**Results:**

A total of 116 differentially expressed MRGs were detected in the TCGA dataset. We found that 12 MRGs were most significantly associated with OS by using the univariate regression analysis in LUAD. Then, multivariate Cox regression analyses were applied to construct the prognostic signature, which consisted of six MRGs-aldolase A (ALDOA), catalase (CAT), ectonucleoside triphosphate diphosphohydrolase-2 (ENTPD2), glucosamine-phosphate N-acetyltransferase 1 (GNPNAT1), lactate dehydrogenase A (LDHA), and thymidylate synthetase (TYMS). The prognostic value of this signature was further successfully validated in the GSE31210 dataset. Furthermore, the calibration curve of the prognostic nomogram demonstrated good agreement between the predicted and observed survival rates for each of OS. Further analysis indicated that this signature could be an independent prognostic indicator after adjusting to other clinical factors. The high-risk group patients have higher levels of immune checkpoint molecules and are therefore more sensitive to immunotherapy. Finally, we confirmed six MRGs protein and mRNA expression in six lung cancer cell lines and firstly found that ENTPD2 might played an important role on LUAD cells colon formation and migration.

**Conclusions:**

We established a prognostic signature based on MRGs for LUAD and validated the performance of the model, which may provide a promising tool for the diagnosis, individualized immuno-/chemotherapeutic strategies and prognosis in patients with LUAD.

**Supplementary Information:**

The online version contains supplementary material available at 10.1186/s12935-021-01915-x.

## Background

Lung cancer is one of the most commonly diagnosed cancer types with high mortality worldwide in men and women [1]. Lung adenocarcinoma (LUAD), which is considered a highly molecular heterogeneous disease, is a prevalent pathological subtype of lung cancer with an average 5-year survival rate of only 15 % [2–4]. Molecular targeted therapy for LUAD has been widely accepted in recent years, and the epidermal growth factor receptor (EGFR) gene, the anaplastic lymphoma kinase (ALK) gene, and the Kirsten rat sarcoma viral oncogene (KRAS) gene are important targets of LUAD [5–7]. Despite great clinical improvements in the molecular basis, diagnosis and treatment modalities of LUAD, the recurrence rate still remains high, and the survival rate remains poor [4, 8]. As LUAD has the tendency of early metastasis, and most of them are found at advanced stages, which may be the most important cause of high mortality in LUAD patients [9, 10]. There is an urgent need, therefore, to develop more reliable and more effective biomarkers for the early detection, diagnosis, prognosis and monitoring of LUAD.

Dysregulated metabolism has been recently identified as a well-recognized hallmark of cancer biology, meeting the requirements of rapid proliferation and preferential survival of cancer cells [11, 12]. In the 1920 s, Otto Warburg first discovered that cancer cells vigorously take up glucose and convert pyruvate into lactate despite the presence of sufficient oxygen, a phenomenon now widely termed aerobic glycolysis, or the Warburg effect [13, 14]. This phenomenon not only provide a niche for the survival and proliferation of tumor cells, but also has a profound effect on the tumor microenvironment [15]. In addition, it has recently been reported that high concentrations of lactate in the tumor microenvironment are associated with distant metastasis and poor prognosis in a multitude of cancers, including LUAD [16, 17]. There is general agreement that the metabolic processes play an important role in the pathogenesis and progression of lung cancer. However, few studies have comprehensively analyzed the relationship between metabolism-related genes (MRGs) and the diagnosis, risk stratification, prognosis, and survival of LUAD by high-throughput biomarker sequencing.

In the present study, we constructed a prognostic signature based on MRGs from The Cancer Genome Atlas (TCGA) database, which was further validated in the GSE31210 dataset to explore an efficient metabolic biomaker for the more accurate stratification management of LUAD. In addition, a nomogram that combined six MRGs was created for predicting the 1-, 3- and 5-year OS of LUAD. The accuracy of the nomogram prediction was evaluated using a calibration plot. Cox regression analysis was applied to identify the prognostic value and clinical relationship of the signature in LUAD. Moreover, the high-risk group patients have higher expression of immune checkpoint molecules and are more sensitive to immunotherapy. Finally, we confirmed six MRGs protein and mRNA expression in six lung cancer cell lines and firstly found that ENTPD2 might played an important role on LUAD cells colon formation and migration.

## Materials and methods

### Data collection

The transcriptomic and the corresponding clinical data of patients with LUAD were downloaded from TCGA (https://portal.gdc.cancer.gov/) database and the Gene Expression Omnibus (GEO; https://www.ncbi.nlm.nih.gov/geo/) database. The RNA-seq data, including 497 LUAD and 54 adjacent non-tumor cases from TCGA database and 174 LUAD cases from GSE31210 dataset were examined. The MRGs were identified from the metabolic pathway-related gene sets of “c2.cp.kegg.v7.0.symbols” in gene set enrichment analysis (GSEA). MRGs can be further analyzed only when they are included in the above data sets.

### Differentially expressed MRGs and enrichment analysis

The differentially expressed MRGs in LUAD and normal lung tissues were detected using the R package limma and the Wilcoxon test method [18]. |logFC|>1 and adjusted *P* < 0.05 were considered as significant. To explore the characteristic biological function and potential pathways of these MRGs, Gene Ontology (GO) and Kyoto Encyclopedia of Genes and Genomes (KEGG) pathway enrichment analysis were were carried out with R package clusterProfiler [19]. Functional categories with a false discovery rate (FDR) smaller than 0.05 were presented.

### Construction of the metabolism‑related signature for LUAD

To avoid the interference of irrelevant factors, patients with follow-up time between 0 day and 2000 days were included. The 426 LUAD samples with survival information in the TCGA dataset were taken as the training set for constructing the prognosis risk model, and the 174 LUAD samples with survival information in the GSE31210 dataset were explored for external validation. Firstly, univariate Cox analysis was used to screen out MRGs associated with the OS of patients with LUAD, and only MRGs with a *P* value < 0.001 were selected for subsequent analyses. To avoid the prognostic signature overfitting and narrow the genes for prediction of the OS, Lasso Cox regression was carried out using R “glmnet” package. MRGs detected via Lasso algorithm were evaluated by step wise multivariate cox regression analysis. By weighting the estimated cox regression coefficients, the model of tumor-related metabolism genes risk was constructed [20]. The prognostic metabolism-related gene signatures were shown as risk score = Ʃ (βi × Expi), where βi, the coefficients, represented the weight of the respective signature and Expi represented the expression value. Based on the risk score formula, patients were assigned into low-risk group and high-risk group with the median risk score as the cut-off point. The Kaplan-Meier (K-M) survival curve was used the log-rank test to evaluate the differences in survival rate between the two groups. Furthermore, the receiver operating characteristic (ROC) curve was implemented by R “survival ROC” package [21] and the corresponding area under the ROC curve (AUC) was measured to assess the sensitivity and specificity of the metabolism-related signature.

### Validation of the metabolism‐related signature for LUAD

To verify the prognostic value of metabolism-related signature, we used the GSE31210 dataset as the validation cohort. The same formula was used to calculate the risk scores for each patient. Survival and ROC curve analyses were implemented as described above. Finally, according to the results of multivariate cox regression analysis, a nomogram for predicting the likelihood of 1-year, 3-year and 5-year OS was constructed by R “rms” package. The calibration plots were used to evaluate the prognostic accuracy of the nomogram.

### Analysis of these crucial MRGs expression level

Differential expression of these hub MRGs at the transcription level were examined by matching cancer and adjacent normal lung tissues from the TCGA database. For further validation of our analysis, The Human Protein Atlas (HPA) online database (http://www.proteinatlas.org/) was applied to identify the expression of these MRGs at the translational level [22].

### Association of the prognostic signature and clinicopathological features

In addition, univariate and multivariate analyses were used to estimate the effect of risk score on OS and the clinicopathologic features (age, gender, clinical stage and pathological grading). We also explored the correlation between the expression of these MRGs and several clinical features. Time-dependent ROC curve was performed to compare the accuracy of the prediction between the clinicopathologic features and risk score.

### Assessing the immuno-/chemotherapeutic response of the risk subtypes for LUAD patients

Immune checkpoint therapy has made important clinical advances and offered a new weapon against cancer, which would ideally be matched to those patients most likely to benefit [23, 24]. Then, we investigated the expression of crucial immunomodulators between low-risk group and high-risk group. Moreover, according to the Genomics of Drug Sensitivity in Cancer (GDSC, https://www.cancerrxgene.org), the chemotherapeutic response for common chemotherapy drugs of each LUAD patients was calculated by by R “pRRophetic” package[25].

### Colon assay and Western blot analysis

Colon assay and Western blot analysis were performed as described previously [26, 27].

### Quantitative real-time PCR

Total RNA was extracted with the TRIzol Reagent (Invitrogen Carlsbad, CA, USA), and the concentration was measured using an ultraviolet (UV) spectrophotometer (UV-1201; Shimadzu Corporation, Kyoto, Japan). Reverse transcription (RT) was performed as described previously [27]. Real-time PCR was conducted using the SYBR-Green PCR kit (Takara, Osaka, Japan) in a Rotor-Gene 3000 machine (Corbett Life Science, Sydney, Australia). The quantitative analysis of the transcription of CAT, LDHA and ENTPD2 was described previously [27]. Each reaction was performed in a 25µL volume containing 2µL of cDNA, 0.5µL of 10µM per each primer, and 12.5 µL of the 2× SYBR-Green mixture. CAT: For: 5′-AGA TGC GGC GAG ACT TTC-3′, Rev: 5′-CAA CTG GGA TGA GAG GGT AG-3′. LDHA: For: 5′-CTG TAT GGA GTG GAA TGA ATG-3′, Rev: 5′ -GAT GTG TAG CCT TTG AGT TTG-3′. ENTPD2: For: 5′-GAC GCT GGT TCT TCA CAC − 3′, Rev: 5′ -CTC TTT GGG CAC ATC CTG-3′.

### Statistical analysis

All statistical analyses were performed by version 3.6.1 of R software (https://www.r-project.org/) and version 5.28.1 of Perl software (http://www.perl.org). The Wilcoxon test was used to compare two paired groups. The Kaplan-Meier survival curves were compared with the log-rank test. If not otherwise stated, data were considered to be statistically significant with *P* value < 0.05.

## Results

### Identification of differentially expressed MRGs in LUAD

**Fig. 1:**
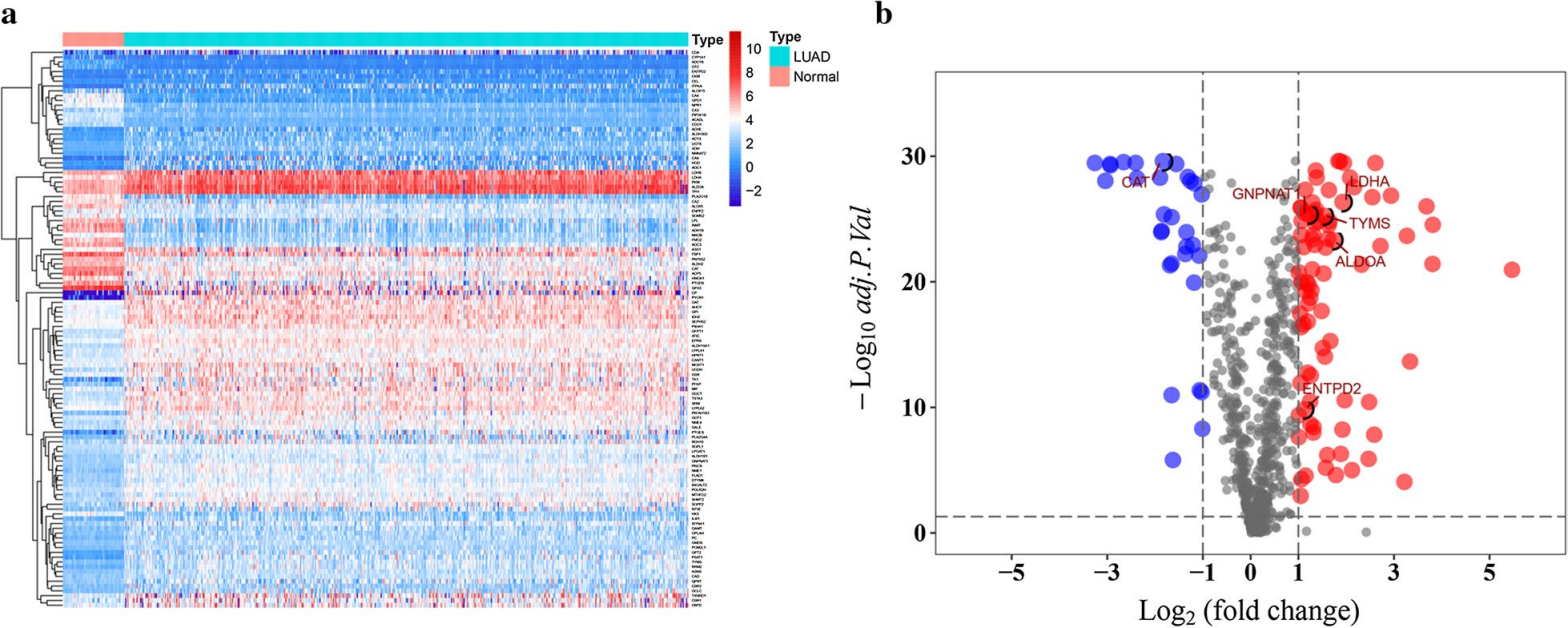
Differentially expressed MRGs in LUAD. **a** Heatmap of MRGs between LUAD and normal lung tissues in TCGA database; The color from blue to red represents the progression from low expression to high expression. **b** Volcano plot of MRGs in TCGA database; The red dots in the plot represents upregulated genes and blue dots represents downregulated genes with statistical significance. Gray dots represent no differentially expressed genes

According to the KEGG metabolic pathway-related gene sets, a total of 944 MRGs were obtained from the gene sets of “c2.cp.kegg.v7.0.symbols”. We matched these genes with the sequence data of LUAD related mRNA in the TCGA database and GSE31210 dataset, and only common mRNAs were used. Considering the cutoff criteria (adjusted *P* value < 0.05 and |log FC| > 1.0), 116 differentially expressed MRGs, which consist of 31 downregulated and 85 upregulated MRGs (Fig. 1), were selected for subsequent analysis.

### Functional enrichment of the differentially expressed MRGs

**Fig. 2:**
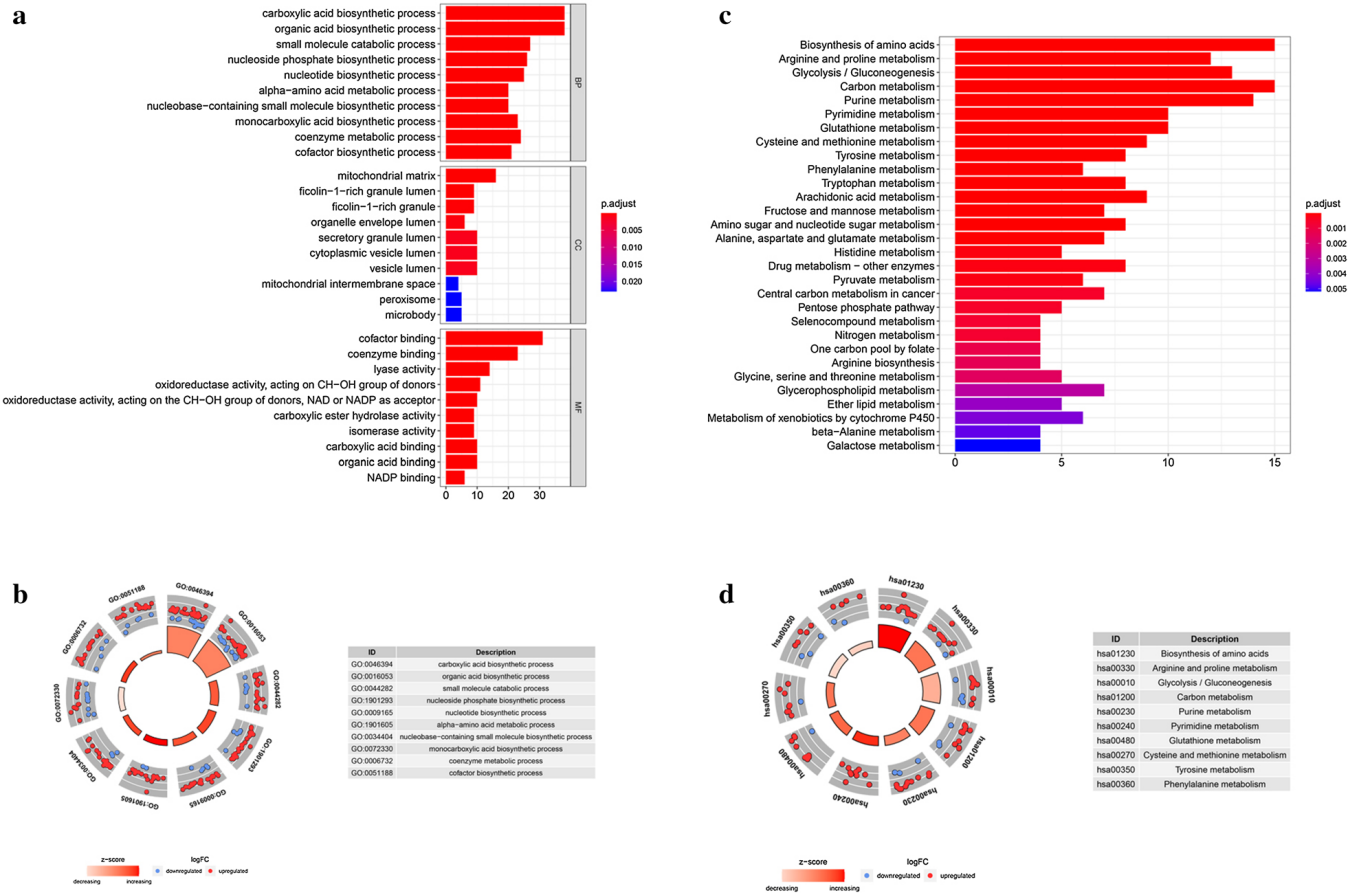
Gene functional enrichment analysis of differentially expressed MRGs. **a** The top 30 significant terms of GO function enrichment. BP biological process, CC cellular component, MF molecular function. **b** The GO circle shows the scatter map of the logFC of the specified gene. **c** The top 30 significant terms of KEGG analysis. **d **The KEGG circle shows the scatter map of the logFC of the specified gene. The higher the Z-score value indicated, the higher expression of the enriched pathway

To investigate the potential functional implication of these MRGs, 116 differentially expressed MRGs were further analyzed by GO functional enrichment analysis and KEGG pathway enrichment analysis. A total of 431 GO terms and 42 pathways were identified (adjusted *P* < 0.05). The top 30 enrichment GO analysis and top 30 enrichment KEGG analysis were displayed in Fig. 2. The top enriched GO terms in biological processes were carboxylic acid biosynthetic process and organic acid biosynthetic process, and those in cellular components were mitochondrial matrix, ficolin-1-rich granule lumen, and ficolin-1-rich granule, in terms of molecular function, genes were mostly enriched in terms of co-factor binding. In the KEGG pathway enrichment analysis, these genes were shown to be significantly associated with signaling pathway related to material synthesis and material metabolism, such as “biosynthesis of amino acids”, “arginine and proline metabolism”, “glycolysis/gluconeogenesis”, “carbon metabolism” and et al.

### Establishment of metabolism‐related prognostic signature for LUAD

**Fig. 3:**
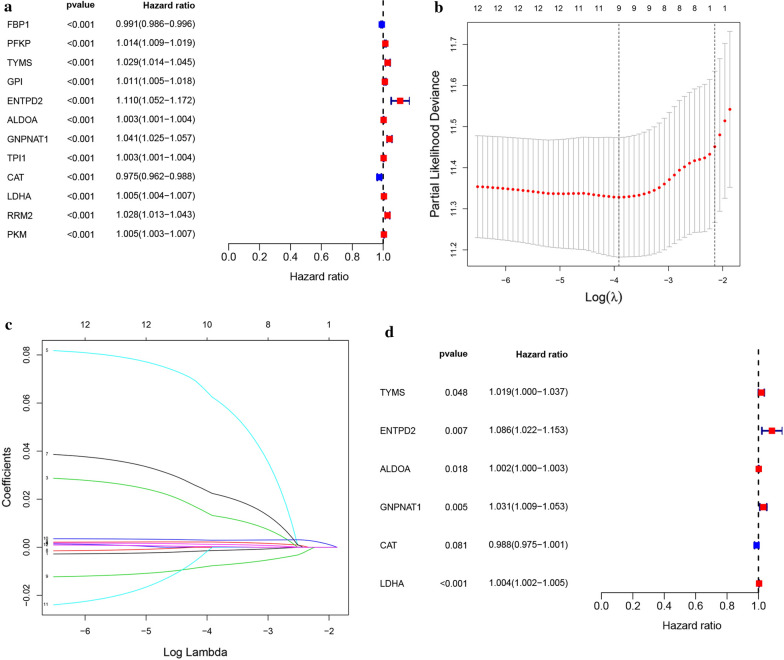
Establishment of metabolism-related prognostic signature . **a** Identified these differentially expressed MRGs related to the LUAD risks by univariate cox regression analysis. *P* values < 0.001 were considered to be statistically significant. **b** Screening of optimal parameter (lambda) at which the vertical lines were drawn. **c** Lasso coefficient profiles of the seventeen MRGs with non-zero coefficients determined by the optimal lambda. **d** Multivariate cox analysis to developing a prognostic index based on these MRGs

To identify MRGs associated with OS, a univariate cox proportional hazard regression analysis was initially performed on 116 differentially expressed MRGs in the TCGA database. The result showed that 12 MRGs were significantly associated with the OS (Fig. 3a; *P* < 0.001). Of the survival-related MRGs, 10 genes (ALDOA, TPI1, PKM, LDHA, GPI, PFKP, RRM2, TYMS, GNPNAT1, and ENTPD2) were considered risk factors (all *P* < 0.001; HRs, 1.0026–1.1103) and that their overexpression might reduce survival. While, overexpression of the remaining two genes (CAT and FBP1) (all *P* < 0.001; HRs,0.9747 and 0.9907, respectively) might improve the survival of patients. The Lasso regression analysis was then used to remove MRGs that may be highly related to other MRGs (Fig. 3b–c). Furthermore, a prognostic signature model was established based on multivariate Cox regression analysis. Finally, six MRGs were confirmed and applied to establish a metabolism-related signature (Fig. 3d). A prognostic model was constructed to evaluate the prognosis of each patient as follows: Risk score = (0.001709×expression value of ALDOA) + (-0.01187×expression value of CAT) + (0.082279×expression value of ENTPD2) + (0.030344×expression value of GNPNAT1) +(0.003499×expression value of LDHA) + (0.018476×expression value of TYMS).

**Fig. 4:**
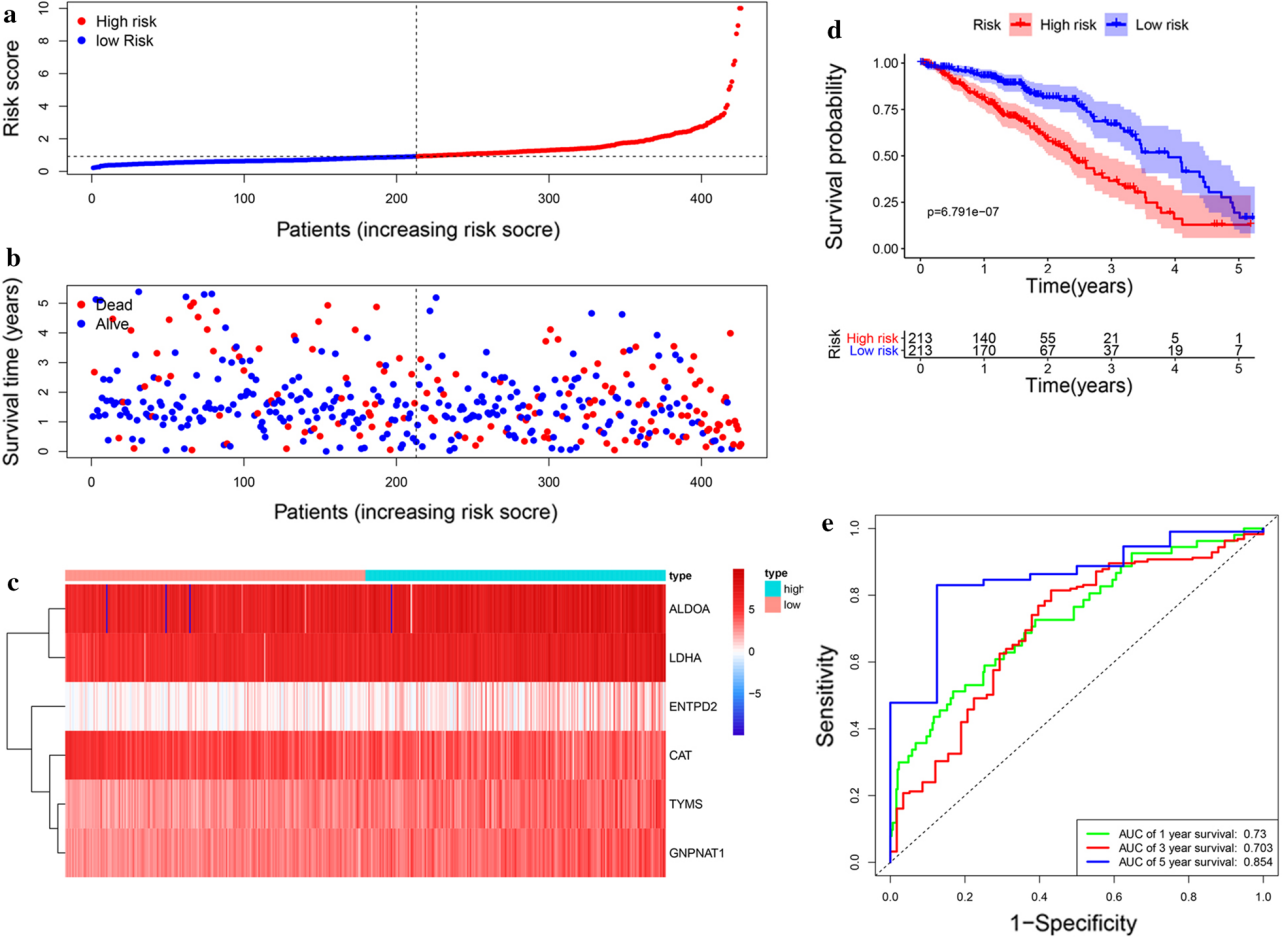
Construction of the metabolism-based prognostic risk signature in the TCGA cohort. **a **The risk score distribution of LUAD patients; **b **Survival status and duration of patients; **c** Heatmap of the metabolism-related genes expression; **d** Survival curves for the low risk and high risk groups; **e** Time-independent receiver operating characteristic (ROC) analysis of risk scores for prediction the OS in the TCGA dataset

Then, the risk score of each patient was calculated according to this prognostic model. Based on the median risk score, 426 LUAD patients were classified into a high risk group (n = 213) and low risk group (n = 213). The risk score, survival status and gene expression heatmap of these prognostic MRGs are presented in Fig. 4a–c. Kaplan-meier log-rank test indicated that patients in the high risk group showed markedly poorer OS than those in the low risk group (Fig. 4d). Areas under the curve value of the signature predicting the 1-, 3- and 5-year OS rates were 0.73, 0.703 and 0.854, indicating that this prognostic model exhibited a good sensitivity and specificity (Fig. 4e).

### Validation of the metabolism‐related prognostic signature for LUAD

**Fig. 5:**
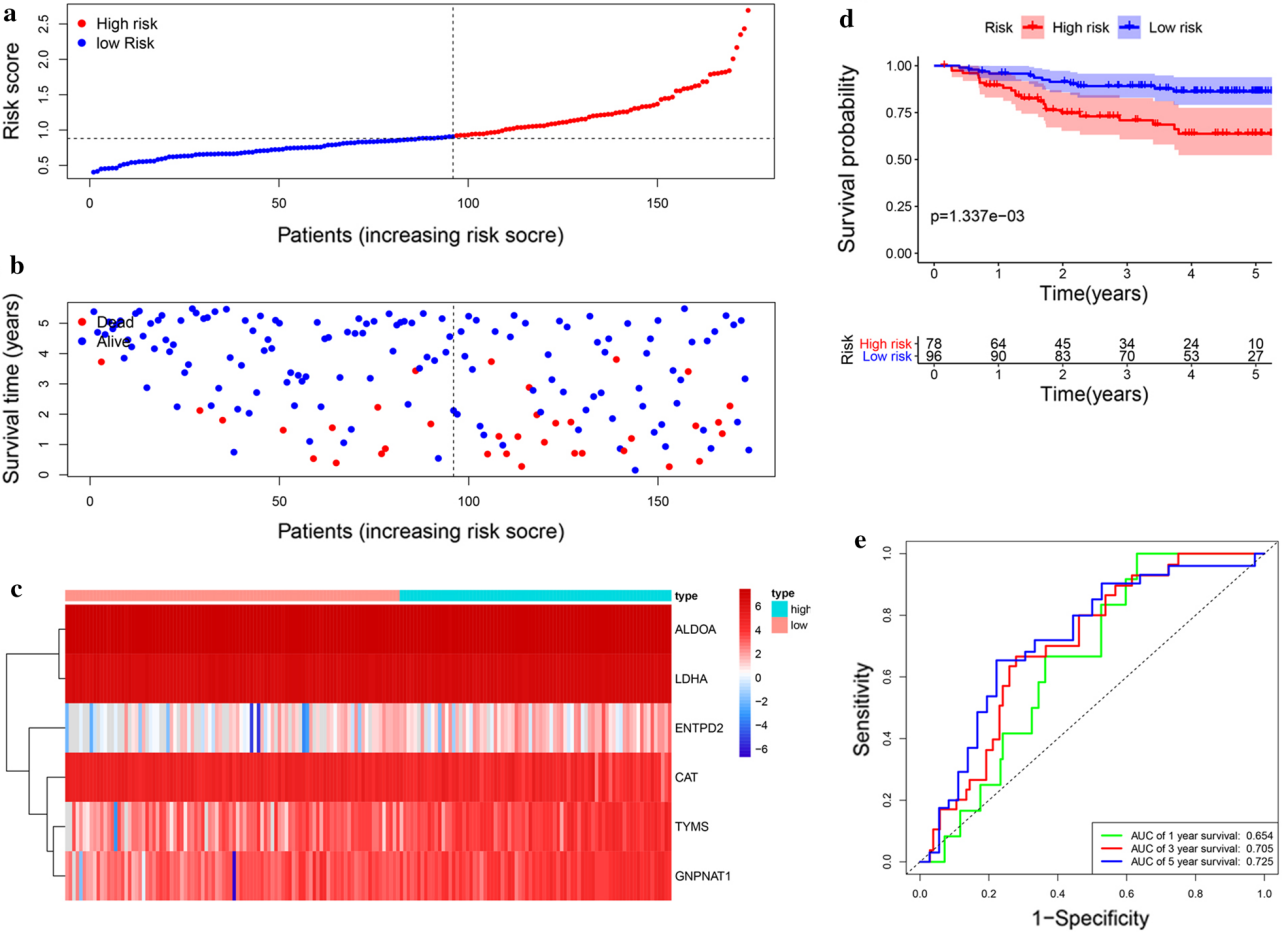
Validation of the metabolism-based prognostic risk signature in the GSE31210 cohort. **a **The risk score distribution of LUAD patients; **b **Survival status and duration of patients; **c **Heatmap of the metabolism-related genes expression; **d **Survival curves for the low risk and high risk groups; **e **Time-independent ROC analysis of risk scores for prediction the overall survival in the GSE31210 dataset

**Fig. 6:**
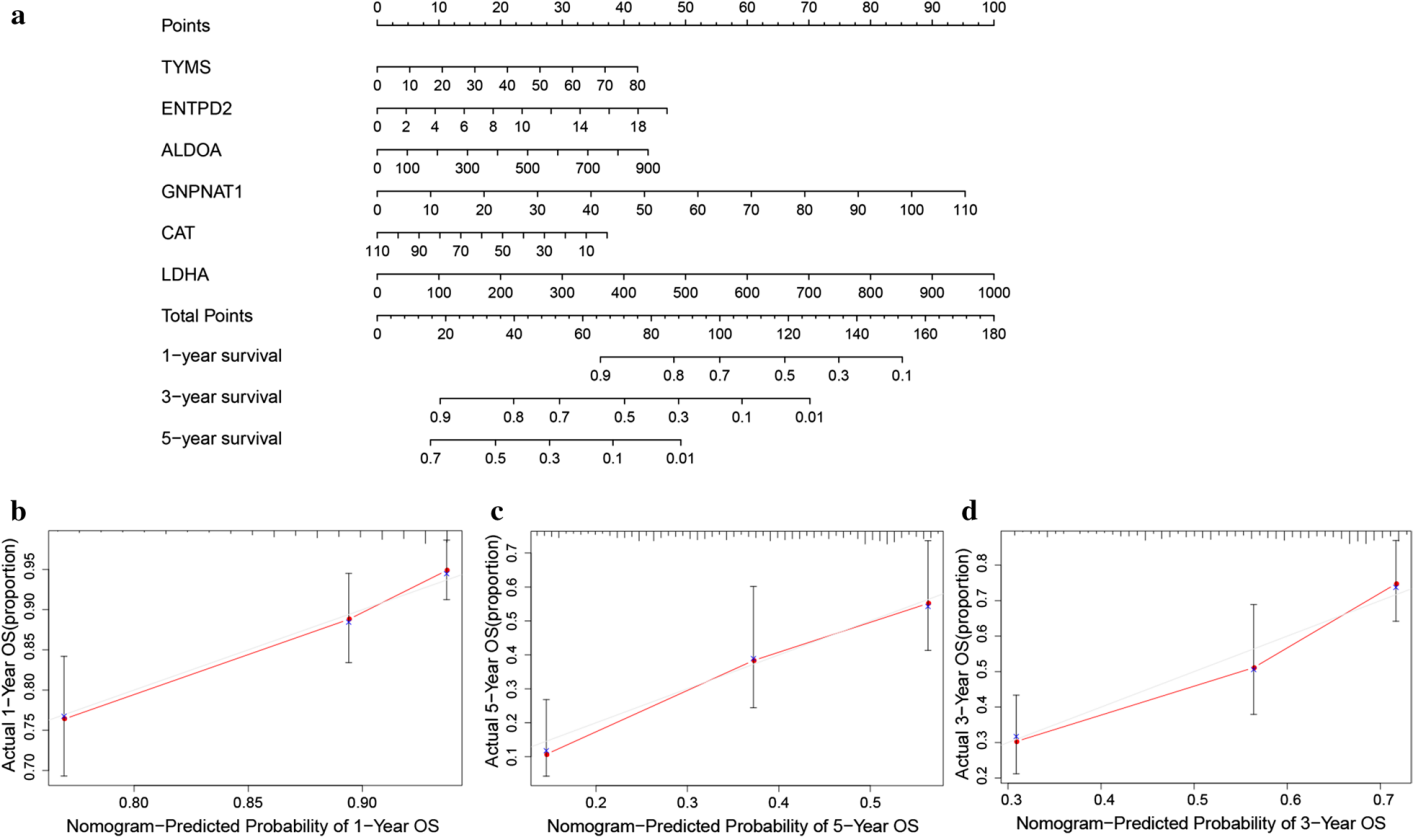
Construction of a nomogram based on the metabolism-related signature in the TCGA cohort. **a** The nomogram based on the signature in LUAD patients at 1, 3, and 5 years. **b–d** Calibration curves of nomogram for the signature at 1, 3, and 5 years

The GSE31210 dataset including 174 LUAD samples were used for the validation of the metabolism-related signature. According to the median risk score, we divided patients into high risk (n = 78) and low risk groups (n = 96). Consistent with the results derived from the TCGA database, the Kaplan-Meier curve demonstrated that patients in the high risk group exhibited markedly poorer OS than those in the low risk group (Fig. 5d). The risk score, survival status and gene expression heatmap of these prognostic MRGs were shown in Fig. 5a-c. The AUCs for 1-, 3- and 5-year OS rates were 0.654, 0.705 and 0.725 (Fig. 5e). A nomogram for predicting 1-, 3- and 5-year OS of patients with LUAD was constructed with the six prognostic genes that had most significant values in multivariate analysis (Fig. 6a). In addition, the calibration curve of the prognostic nomogram demonstrated good agreement between the predicted and observed survival rates for each of OS (Fig. 6b-d).

### Analysis of these crucial MRGs expression level

**Fig. 7:**
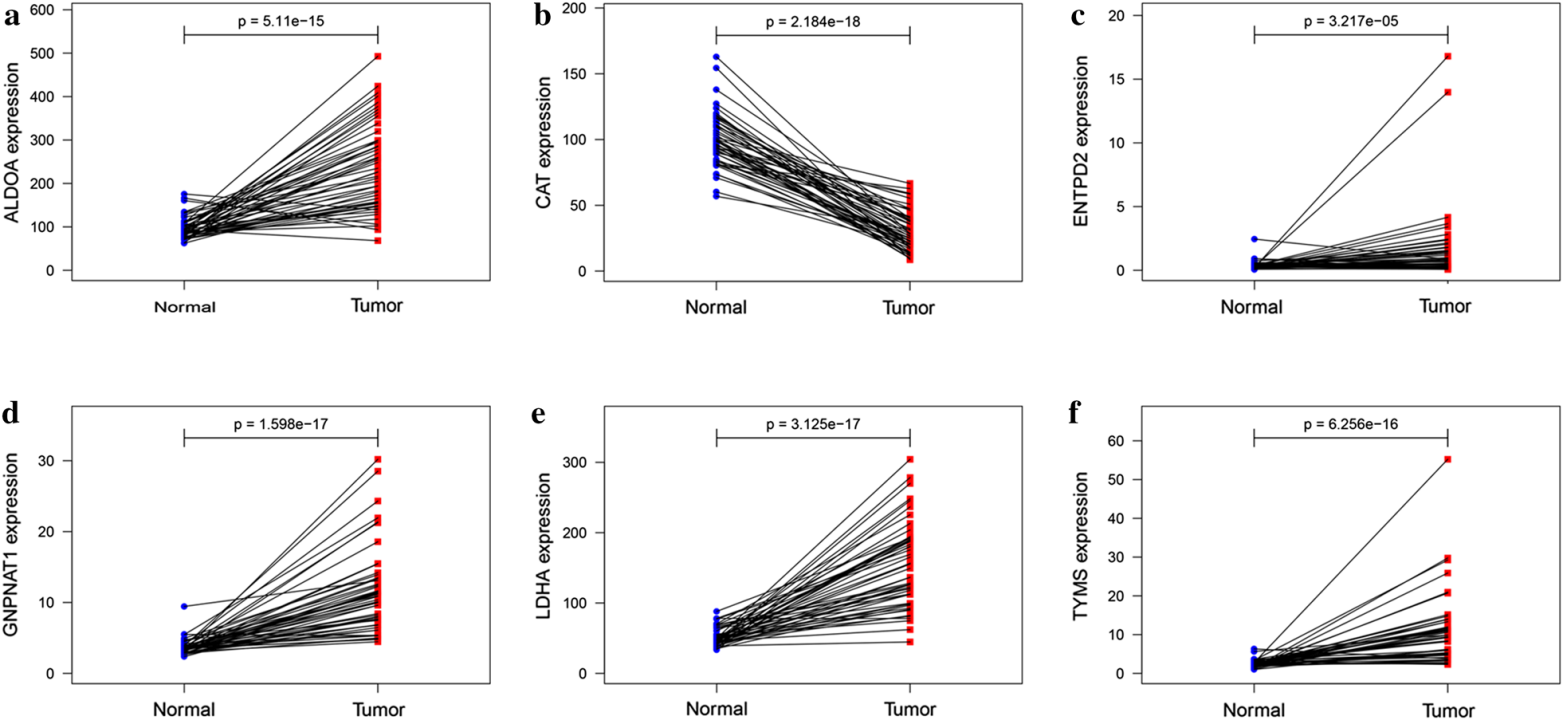
Comparison of the crucial genes mRNA levels in paired adjacent normal tissues and LUAD tissues from TCGA. **a** ALDOA, **b **CAT, **c** ENTPD2, **d** GNPNAT1, **e **LDHA, **f **TYMS

**Fig. 8:**
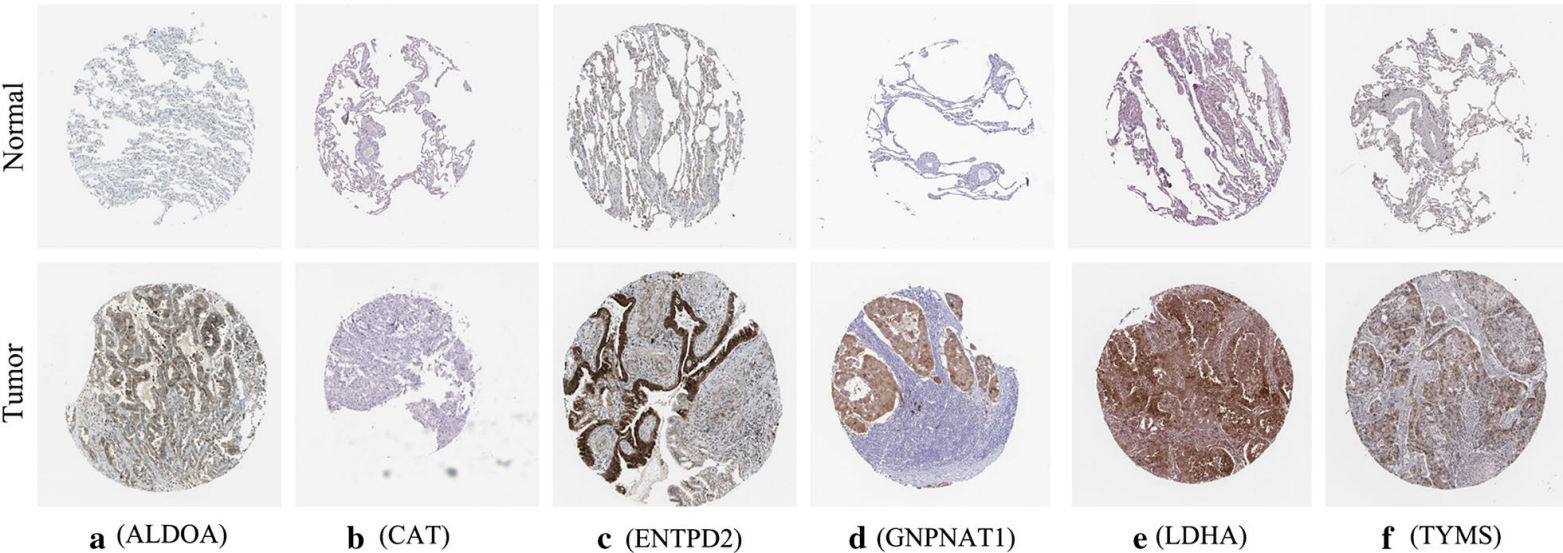
Verification of hub MRGs expression in LUAD and normal lung tissue using the HPA database. **a** ALDOA, **b **CAT, **c** ENTPD2, **d** GNPNAT1, **e** LDHA, **f **TYMS

To explore the six hub genes at the transcription level, the mRNA expression levels were analyzed using the TCGA database. The results demonstrated that the expression of ALDOA, ENTPD2, GNPNAT1, LDHA, and TYMS in LUAD tissues were all increased than those of adjacent normal lung tissues, while the expression of CAT was decreased than those of adjacent tissues (Fig. 7). Subsequently, we also investigated the expression of hub genes between low and high risk group. The results showed that high risk group had higher expression of five hub MRGs (ALDOA, ENTPD2, GNPNAT1, LDHA, and TYMS) than low risk group, while the expression of CAT was decreased than high risk group (Additional file 1: Figure S1, *P* < 0.001). To assess the six hub genes at the translational level, the protein expression levels were analyzed using the HPA database. The results showed that the protein level of ALDOA, ENTPD2, GNPNAT1, LDHA, and TYMS were increased in LUAD tissues than in normal tissues, matched their mRNA expression levels (Fig. 8). There is no difference between LUAD tissues and normal tissues for the protein level of CAT (Fig. 8b). Finally, to further investigate functional implication of six MRGs, GO and KEGG analysis were conducted in R software. GO analysis showed that six MRGs were mainly enriched in metabolic process, including purine nucleoside diphosphate, puriner ibonucleoside diphosphate, ribonucleoside diphosphate and nucleoside diphosphate metabolic process (Additional file 2: Figure S2a). KEGG analysis showed that six MRGs were significantly enriched glycolysis/gluconeogenesis, HIF-1 signaling pathway and carbon metabolism (Additional file 2: Figure S2b).

### Clinical value of prognostic signature

**Fig. 9:**
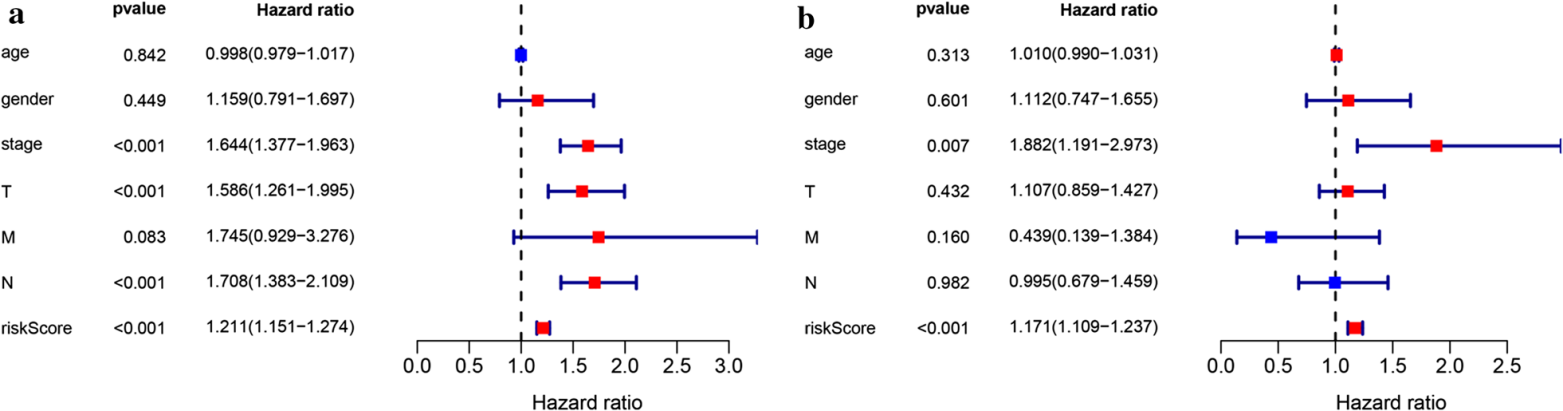
Univariate (**a**) and multivariate (**b**) independent prognostic analysis of independent risk factors for OS in patients with LUAD

**Fig. 10:**
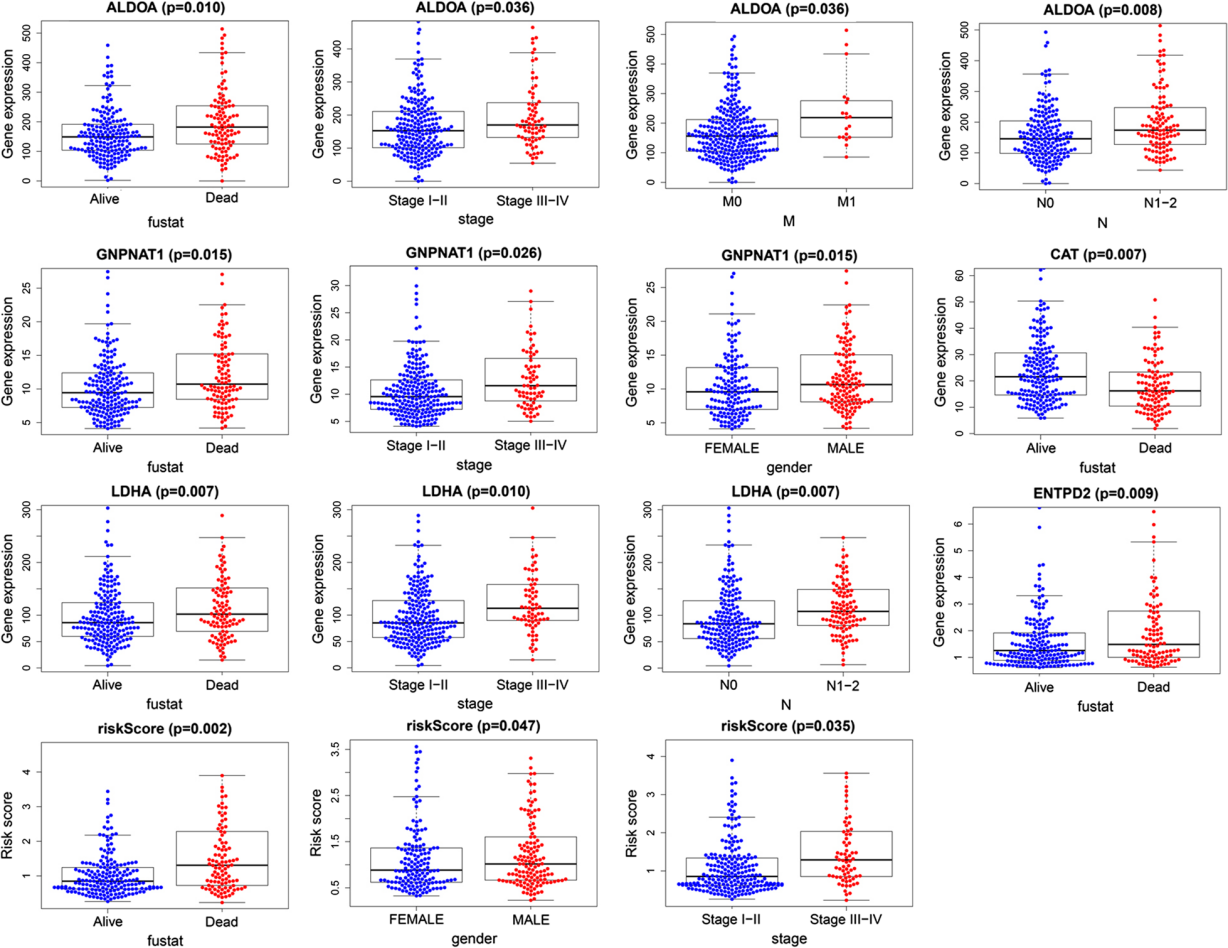
Relationships between MRGs expression and clinicopathological factors in LUAD (*P* < 0.05)

Univariate and multivariate Cox regression analysis was conducted to evaluate the independent prediction ability of metabolism-related prognostic signature between the signature and other common prognostic factors, including age, gender, histological grade, pathological stage and TNM stage. Although univariate cox analysis indicated that pathologic stage, T stage, N stage and our model were markedly associated with OS (Fig. 9a; *p* < 0.001), after the multivariate analysis, only metabolism-related prognostic signature (*p* < 0.001) and pathological stage (*p* < 0.007) could be used as an independent prognostic factor (Fig. 9b). To further evaluate the clinical value of MRGs, the relationship between MRGs prognostic indicators and clinical features were investigated, and the results indicated that ALDOA, ENTPD2, GNPNAT1, LDHA, and CAT were differentially expressed in patients with various clinical features (Fig. 10). To validate the clinical value of the metabolism-related prognostic signature, the association between the risk score and clinical characteristics were subsequently assessed, and the results demonstrated that high risk scores were positively associated with survival status, gender, N stage, and pathologic stage in patients with LUAD (Fig. 10). To investigate more additional prognostic value of the prognostic signature, time-dependent ROC curve was performed. The AUC value of the risk score predicting the 0.5-, 1-, 2-, 3- and 5-year OS rates were 0.745, 0.779, 0.726, 0.716, 0.760 and 0.784, respectively, which was much higher than clinicopathologic features(Additional file 3: Figure S3). The results revealed that prognostic accuracy of the signature was superior to clinical risk factors.

### Assessment of the immuno-/chemotherapeutic response in the risk subtypes for LUAD patients

**Fig. 11:**
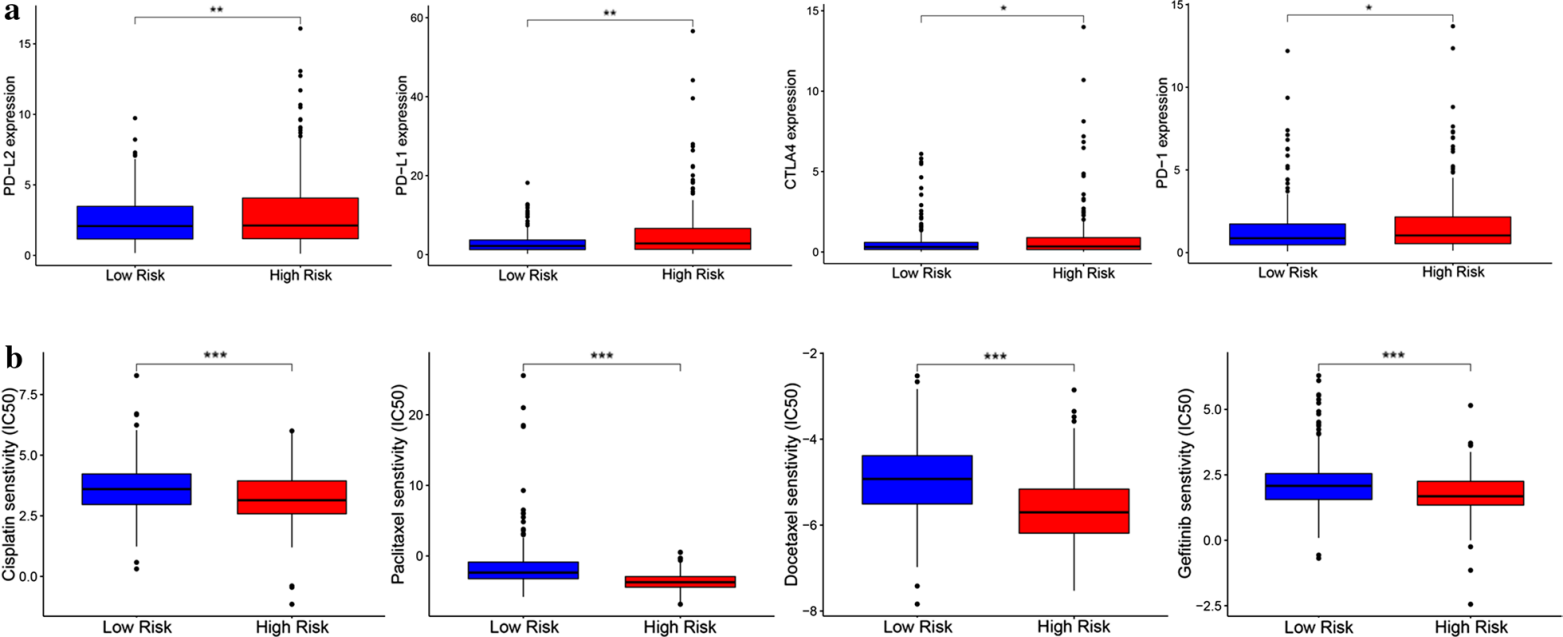
Assessing the immuno-/chemotherapeutic response of the risk subtypes for LUAD patients. **a** The expression of immune checkpoint molecules (PD-L1, PD-L2, CTLA-4 and PD-1) between low-risk group and high-risk group; **b** The IC50 indicated the efficiency of chemotherapy to low- and high-risk groups by cisplatin, paclitaxel, docetaxel and gefitinib. * *p* < 0.05, ** *p* < 0.01, *** *p* < 0.001

Immune checkpoint therapy, which improved antitumor immune responses by regulating the activity of T cells, has been emerged as a new weapon against cancer[24]. Thus, we analyzed the expression of immune checkpoint molecules (PD-L1, PD-L2, CTLA-4 and PD-1) between low-risk group and high-risk group in LUAD samples. As shown in Fig. 11a, high-risk patients with LUAD had significantly higher expression of immune checkpoint molecules than low-risk patients. Chemotherapy is an effective treatment for patients with advanced LUAD. The IC50 values of the low-risk and high-risk groups were calculated based on the GDSC data, as shown in Fig. 11b, the results indicated that no chemotherapeutic drugs with significant response sensitivity were found in the high-risk group. The above results demonstrateted that the poor prognosis of high-risk patients might be related to the immunosuppressive microenvironment and chemotherapy resistance. That is to say, the high-risk group patients have higher expression of immune checkpoint molecules and are more sensitive to immunotherapy.

### Experimental validation

**Fig. 12:**
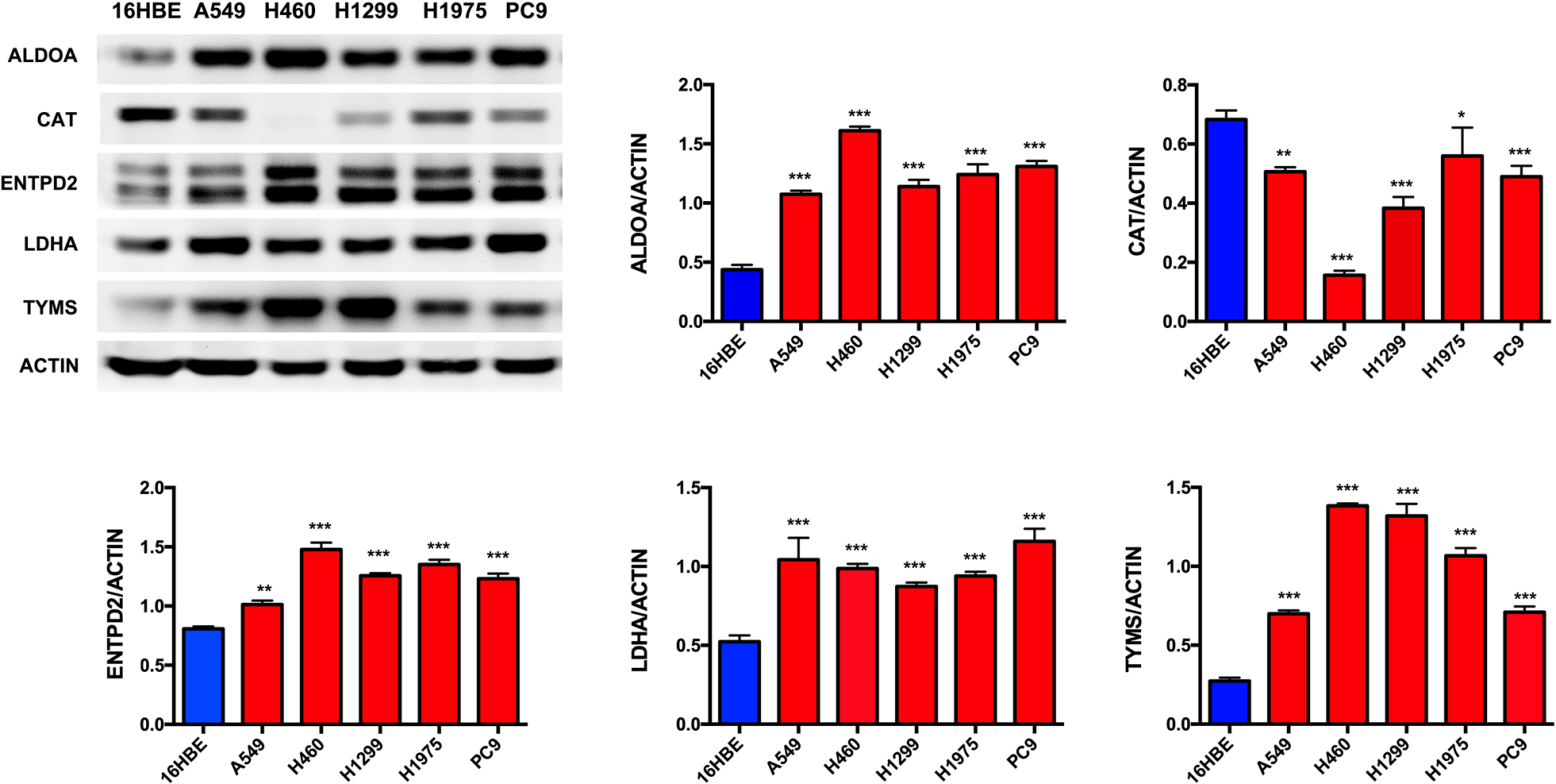
Validation of MRGs protein expression by western blot. * *p* < 0.05, ** *p* < 0.01, *** *p* < 0.001

The protein expression levels of ALDOA, ENTPD2, LDHA, TYMS and CAT were investigated in 5 lung cancer cell lines (A549, H460, H1299, H1975, PC9), normal airway epithelial cells (16HBE) as control. The same with the results we have developed from bioinformatics, ALDOA, ENTPD2, LDHA, TYMS were significantly increased in 5 lung cancer cell line, comparing with in 16HBE. CAT was significantly decreased in 5 lung cancer cell line, comparing with in 16HBE. The gray value of protein bands was quantified (Fig. 12).

**Fig. 13:**
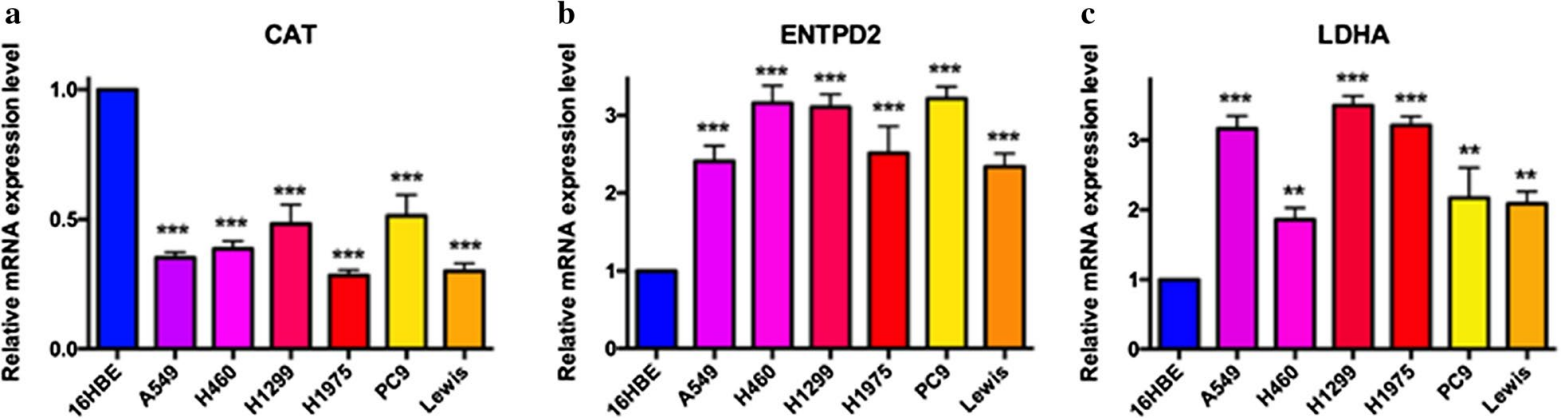
Validation of MRGs mRNA expression by real-time PCR. **a** mRNA expression of CAT in 6 lung cancer cells; **b** mRNA expression of ENTPD2 in 6 lung cancer cells; **c **mRNA expression of LDHA in 6 lung cancer cells; ** *p* < 0.01, *** *p* < 0.001

The mRNA expression levels of CAT, ENTPD2 and LDHA were also investigated in 6 lung cancer cell lines (A549, H460, H1299, H1975, PC9, Lewis), 16HBE as control. The results were also same with protein data, ENTPD2 (Fig. 13a) and LDHA (Fig. 13b) were significantly increased, while CAT (Fig. 13c) was significantly decreased, comparing with in 16HBE.

**Fig. 14:**
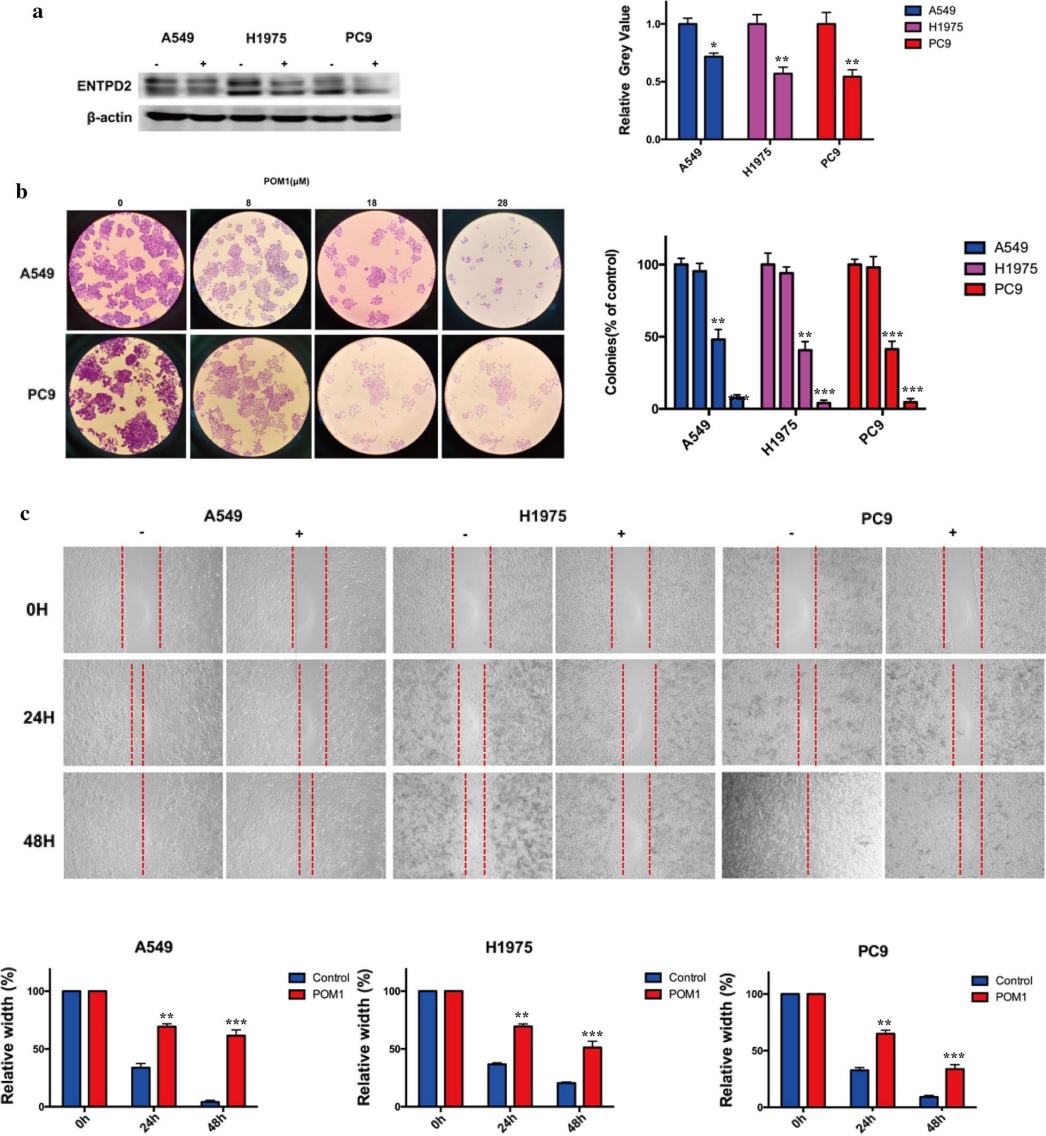
Validation the function of ENTPD2 by colon assay and migration. **a** Confirm of POM1 inhibited ENTPD2 expression by western blot; **b** Inhibit ENTPD2 could inhibit the clone formation in lung adenocarcinoma cells; **c** Inhibit ENTPD2 could inhibit cell migration in lung adenocarcinoma cells. * *p* < 0.05, ** *p* < 0.01, *** *p* < 0.001

Since ENTPD2 may be a good prognostic marker and therapeutic target for cancer patients, especially for those receiving immune therapy [28]. We used ENTPD2 inhibitor POM-1 in 5 lung cancer cell lines, found that it could inhibit the formation of colonies in A549 and PC9, decreased colony-forming was also observed in H1975, which were all lung adenocarcinoma cell lines (Fig. 14b). The protein expression of ENTPD2 in 4 cell lines was confirmed by western blot after adding POM-1 (Fig. 14a). Most importantly, we found POM-1 could inhibit the migration of 5 lung cancer cell lines (Fig. 14c).

## Discussion

LUAD, which is highly heterogeneous in morphological characteristics and remarkably variable in prognosis, is the most prevalent subtype of non-small cell lung cancer (NSCLC) [4, 29]. More and more attention has been recently paid to the key role of gene signatures based on specific correlation in predicting the prognosis of LUAD because of the rapid advances in high-throughput technologies and bioinformatics methodology [30–33]. Moreover, the identification of novel gene signatures that predict the prognosis of patients is beneficial for the choice of treatment regimens and the improvement of survival rate [34, 35].

In recent years, interesting in dysregulated metabolism of cancer has been growing [36]. Accumulating evidence showed that MRGs played a key role in cancer development and progression [37]. Therefore, the identification of novel MRGs has lately become a hotspot in cancer research, both as a biomarker and potential therapeutic target. In this study, a total of 116 differentially expressed MRGs, which consist of 31 downregulated and 85 upregulated MRGs, were detected in the TCGA dataset. We found that 12 MRGs were most significantly associated with OS by using the univariate regression analysis in LUAD. After conducting the LASSO regression and multivariable Cox regression analyses, a novel prognostic signature which consisted of six MRGs (ALDOA, CAT, ENTPD2, GNPNAT1, LDHA, and TYMS) was established. Based on the gene signature, LUAD patients were classified into a high risk group and low risk group. Patients in the high risk group, which had a survival rate lower than 15 %, showed markedly poorer OS than the low risk group. The time-dependent ROC analysis demonstrated that the AUC for 1, 3, and 5 years were 0.73, 0.703, and 0.854, respectively, indicating that this prognostic signature have good sensitivity and specificity. The prognostic value of this signature was further successfully validated in the GSE31210 dataset. Moreover, the calibration curve of the prognostic nomogram demonstrated good agreement between the predicted and observed survival rates for each OS. Further analysis indicated that this signature could be an independent prognostic indicator after adjusting to other clinical factors. Moreover, the high-risk group patients have higher expression of immune checkpoint molecules and are more sensitive to immunotherapy. Finally, the signature was found to be associated with various clinicopathological features.

Furthermore, six genes (ALDOA, CAT, ENTPD2, GNPNAT1, LDHA, and TYMS) in this prognostic signature were selected as crucial MRGs. ALDOA is an important enzyme involved in the glycolysis pathway that is highly expressed in a wide range of cancers [38]. Some studies also proved that the overexpression of ALDOA might contribute to tumorigenesis and the progression of cancers through modulation of HIF-1α signaling [39, 40]. Our results showed that ALDOA might be a tumor-promoting gene in LUAD. Abnormal expression or decreased activity of CAT can lead to an increase in intracellular ROS concentration, which directly or indirectly induces tumorigenesis [41, 42]. Consistently, our study found that compared with normal lung tissues, the mRNA level of CAT in LUAD tissues was down-regulated. GNPNAT1, a member of the GNAT protein superfamily, is a key enzyme in the metabolic pathway of N-acetylglucosamine synthesis [43]. Zhao et al. reported that the overexpression of GNPNAT1 could promote the infiltration and adhesion of lung cancer cells [44]. In line with their findings, we found GNPNAT1 was also increased in LUAD. LDHA catalyzes the conversion of pyruvate to lactate with concomitant oxidation of NADH to NAD+, which plays an essential role in metabolic pathways of the cancer cells [45]. Recently, accumulating evidence showed that the overexpression of LDHA could promote the production of lactate, thus contributing to the acidification of the tumor microenvironment, which may limit the effect of anti-PD-L1 therapy [46, 47]. We also found increased LDHA in LUAD might associated with a poor prognosis. TYMS, a rate-limiting enzyme during the DNA synthesis, plays an important role in catalyzing the methylation of deoxyuridine monophosphate to deoxythymidine monophosphate [48]. High levels of TYMS expression are related to worse responses to 5-FU, shorter survival times and other adverse clinical behaviors in a variety of solid tumors [49, 50]. Since only patients with low expression of TYMS can respond to 5-FU, individualized chemotherapy regimens can be selected according to the expression of TYMS and tumor classification [51]. We also found TYMS as a part of the nomogram could predict LUAD patient prognosis.

In this study, six MRGs prognostic indicators were identified for the first time to be possibly associated with the survival outcome of LUAD. We also confirm the protein and mRNA expression in lung cancer cell lines by some experimental validation. To obtain a deep understanding of the selected genes, the functional annotation analyses of ENTPD2 were performed. ENTPD2 belongs to enzymes nucleoside triphosphate diphosphohydrolase family (NTPDase). NTPDase1(CD39) was played a key role in turning an ATP-mediated immune-stimulating into an adenosine-mediated immunosuppressant tumor microenvironment (TME) involving the coordinated control of inflammatory responses and tumor-associated antigen-specifific T cell immunity [52]. While over-expression of ENTPD2 was a poor prognostic indicator for HCC, ENTPD2 inhibition was able to mitigate cancer growth and enhance the efficiency and efficacy of immune checkpoint inhibitors [28]. In this study, we confirmed ENTPD2 played an important role on cell colon formation and migration in LUAD for the first time.

However, we should acknowledge that there are some limitations in the present study which should be addressed in future studies. First, the potential selection bias could not be ruled out because of the transcriptomic and the corresponding clinical data of patients with LUAD were obtained from public database. Second, the robustness of the prognostic signature must be validated in large prospective studies.

## Conclusions

In summary, we identified a novel signature based on MRGs that could be applied to analyze the prognostic of patients with LUAD, and verified by the data from the GEO databases and experimental validation. Meanwhile, we firstly developed the function of ENTPD2 on cells colon formation and migration in 5 lung cancer cell lines. This signature may provide valuable information either for diagnosis or developing novel therapeutic options for LUAD patients in the future.

## Supplementary Information

**Additional file 1: Figure S1.** The expression of hub MRGs between low and high risk group. *** *p* < 0.001.

**Additional file 2: Figure S2.** GO (a) and KEGG (b) analysis of hub MRGs.

**Additional file 3: Figure S3.** Comparison of time-dependent ROC curves among the age, gender, Pathological_stage, T_stage, M_stage, N_stage, and prognostic signature. **a** 0.5-year OS; **b** 1-year OS; **c** 2-year OS; **d** 3-year OS; **e** 4-year OS; **f** 5-year OS.

## Data Availability

All data generated or analyzed during the present study was downloaded from TCGA database, GEO database, HPA database and GDSC database.

## Abbreviations

TCGA: The Cancer Genome Atlas
GEO: Gene Expression Omnibus
LUAD: Lung adenocarcinoma
GO: Gene Ontology
KEGG: Kyoto Encyclopedia of Genes and Genomes
FDR: False discovery rate
MRGs: Metabolism-related genes
ROC: Receiver operating characteristic
HR: Hazard ratio
CI: Confidence interval
TNM: Tumour size/lymph nodes/distance metastasis, a tumour staging system used in oncology and constructed by the American Joint Committee on Cancer and the Union for International Cancer Control
