# Correction: Establishment and validation of a prognostic signature for lung adenocarcinoma based on metabolism-related genes

**DOI:** 10.1186/s12935-025-04116-y

**Published:** 2025-12-23

**Authors:** Zhihao Wang, Kidane Siele Embaye, Qing Yang, Lingzhi Qin, Chao Zhang, Liwei Liu, Xiaoqian Zhan, Fengdi Zhang, Xi Wang, Shenghui Qin

**Affiliations:** 1 Institute of Pathology, Tongji Hospital, Tongji Medical College Huazhong University of Science and Technology, Wuhan, 430030 China; 2 Department of Pharmacy, Hiser Medical Center of Qingdao, Qingdao, 266033 China; 3 Department of Pathology, Wuhan Third Hospital (Tongren Hospital of Wuhan University), Wuhan, 430030 China

**Correction: Cancer Cell International (2021) 21:219**

10.1186/s12935-021-01915-x.

In this article [1], The Fig. 14.b appeared incorrectly and have now been corrected in the original publication. For completeness and transparency, the old incorrect and correct versions are displayed below. The original article has been corrected.

Incorrect Fig. 14.b:



**Fig. 14**

Validation the function of ENTPD2 by colon assay and migration. **a** Confirm of POM1 inhibited ENTPD2 expression by western blot; **b** Inhibit ENTPD2 could inhibit the clone formation in lung adenocarcinoma cells; **c** Inhibit ENTPD2 could inhibit cell migration in lung adenocarcinoma cells. * *p* < 0.05, ** *p* < 0.01, *** *p* < 0.001

Correct Fig, 14 b:

**Fig. 14:**
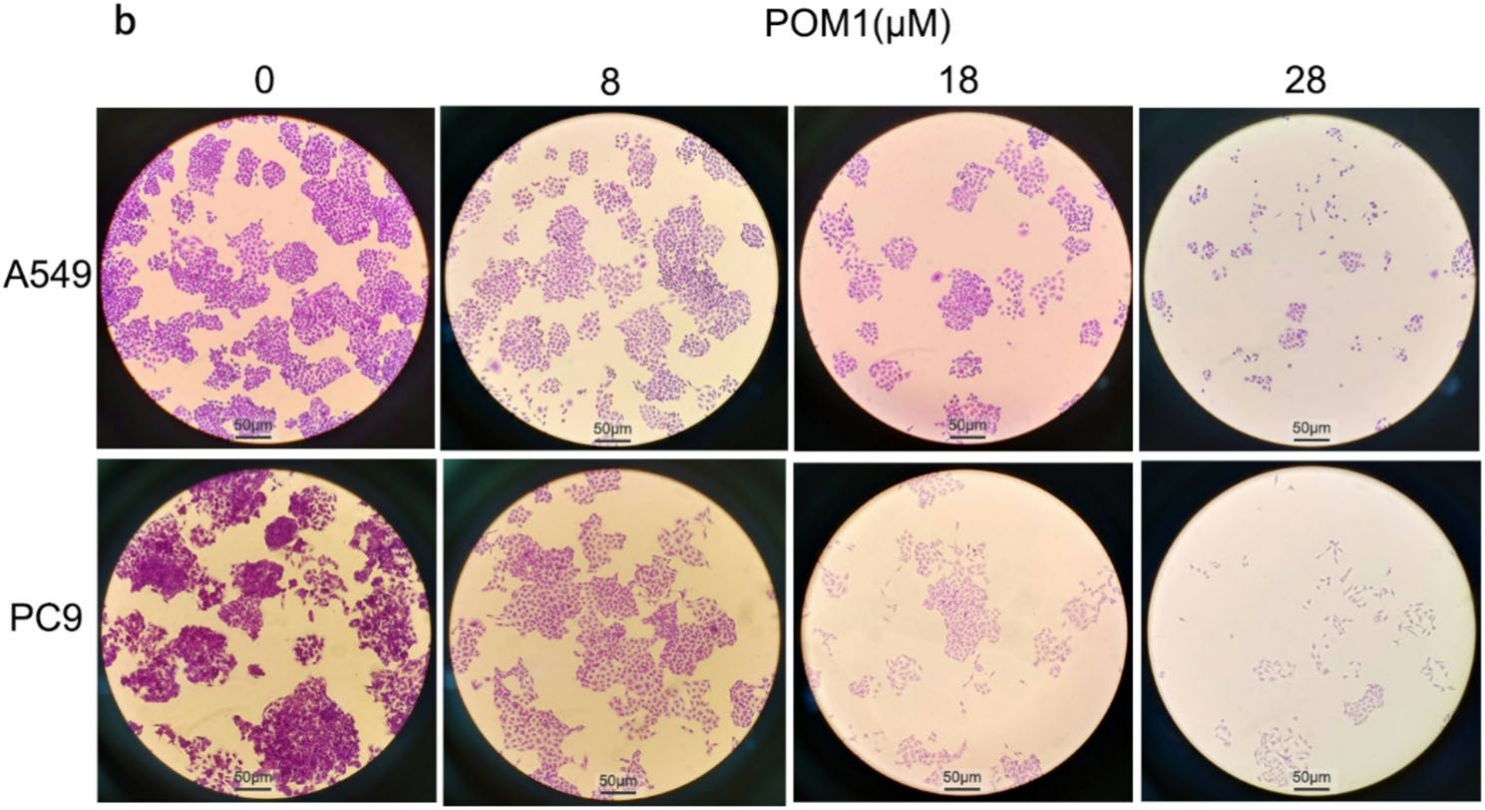
Validation the function of ENTPD2 by colon assay and migration. **a** Confirm of POM1 inhibited ENTPD2 expression by western blot; **b** Inhibit ENTPD2 could inhibit the clone formation in lung adenocarcinoma cells; **c** Inhibit ENTPD2 could inhibit cell migration in lung adenocarcinoma cells. * *p*< 0.05, ** *p *< 0.01, *** *p *< 0.001
