# Supplementary figures and images for: Establishment and validation of a prognostic signature for lung adenocarcinoma based on metabolism‐related genes
Source: Cancer Cell Int. 2021 Apr 15;21:219. doi: 10.1186/s12935-021-01915-x (PMC8050921; doi:10.1186/s12935-021-01915-x)

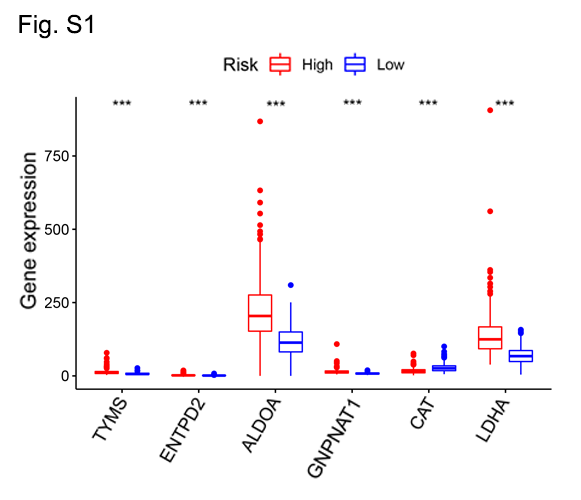

Supplement: Supplementary file 1 — Additional file 1: Figure S1. The expression of hub MRGs between low and high risk group. *** p < 0.001. [file 12935_2021_1915_MOESM1_ESM.tif]

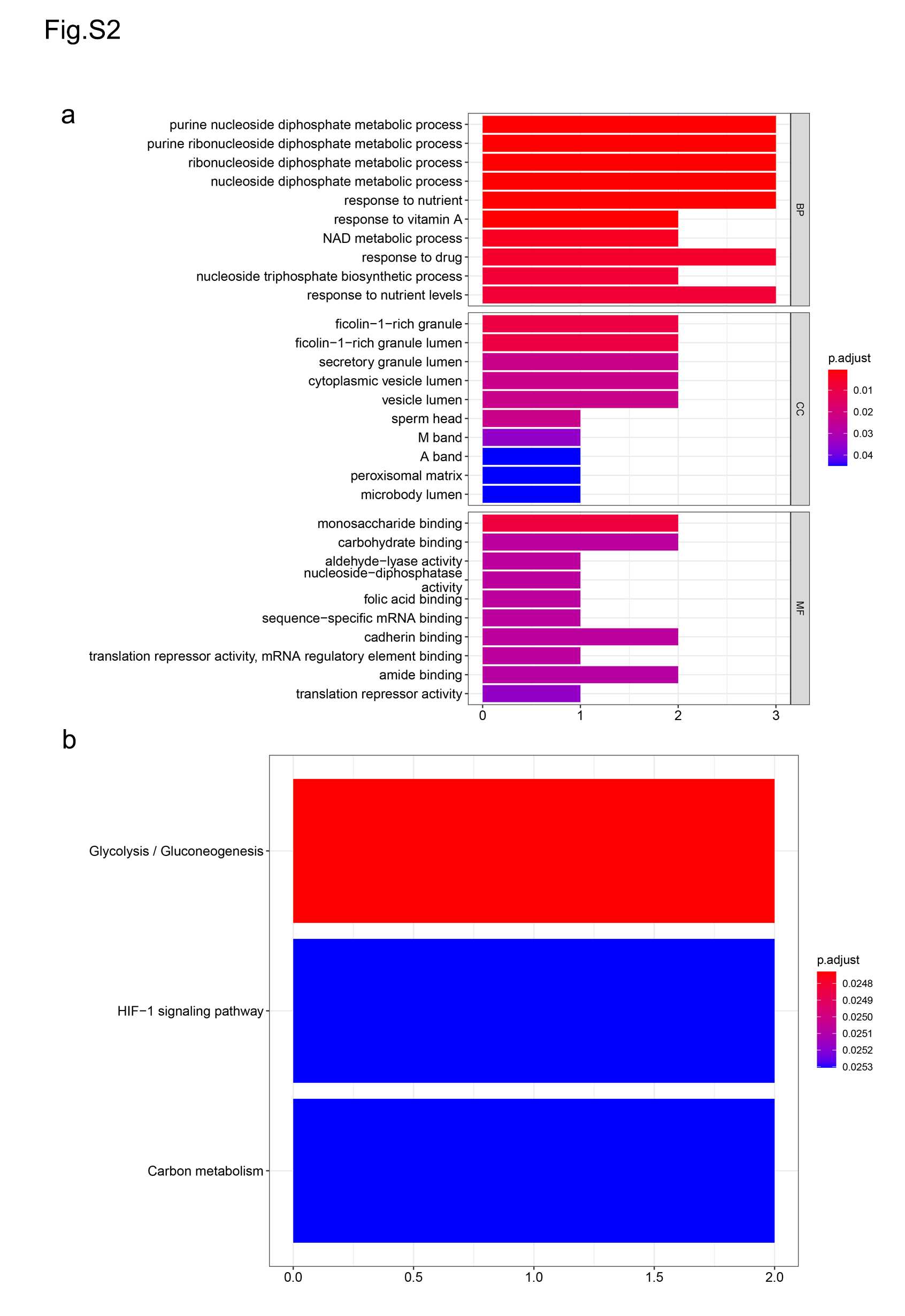

Supplement: Supplementary file 2 — Additional file 2: Figure S2. GO (a) and KEGG (b) analysis of hub MRGs. [file 12935_2021_1915_MOESM2_ESM.tif]

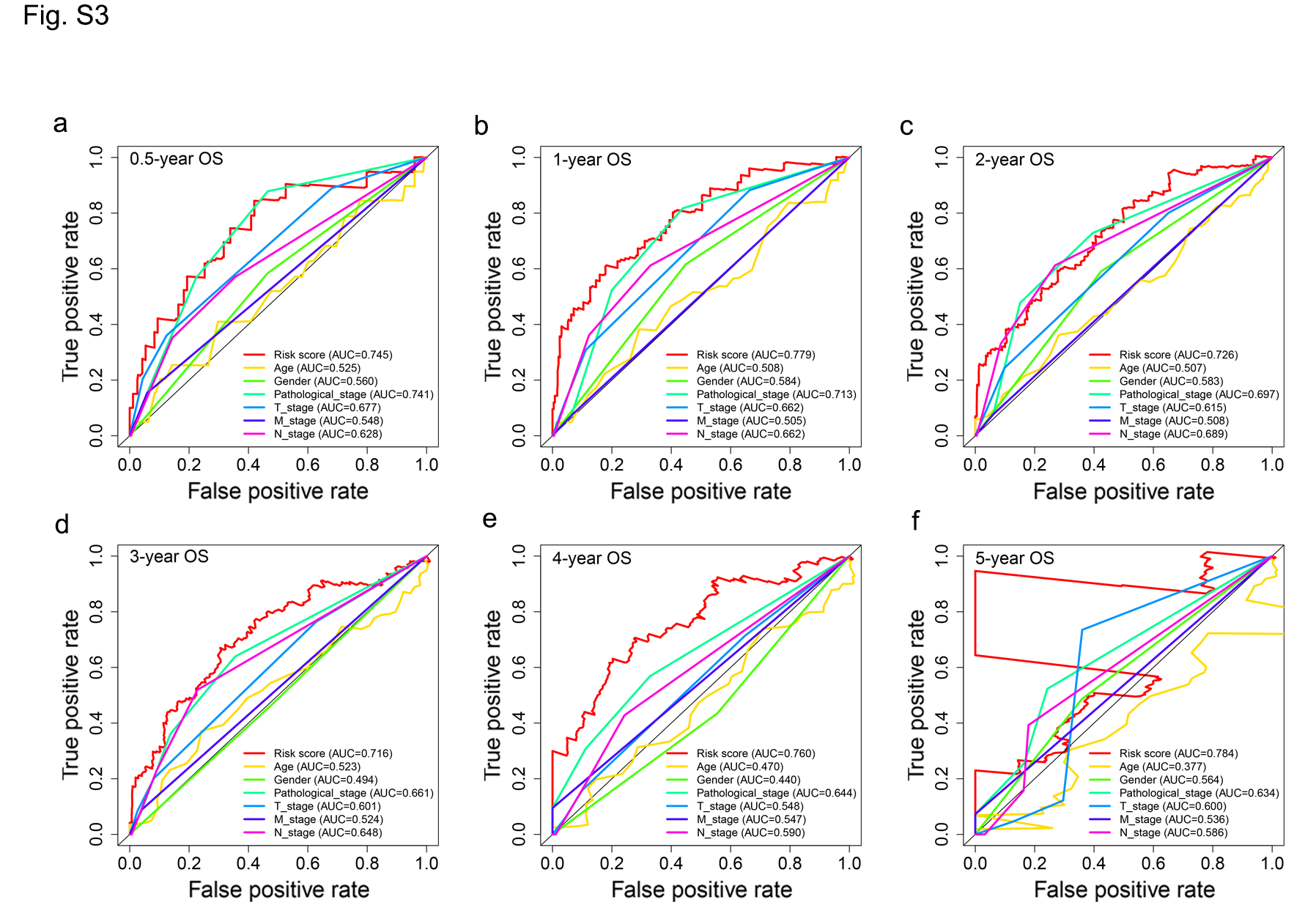

Supplement: Supplementary file 3 — Additional file 3: Figure S3. Comparison of time-dependent ROC curves among the age, gender, Pathological_stage, T_stage, M_stage, N_stage, and prognostic signature. a 0.5-year OS; b 1-year OS; c 2-year OS; d 3-year OS; e 4-year OS; f 5-year OS. [file 12935_2021_1915_MOESM3_ESM.tif]
